# Gastric Duplication Cyst in a man Presenting with Elevated Liver Enzymes and Icterus

**Published:** 2014-03

**Authors:** Seyed Ali Malekhosseini, Farid Moradian

**Affiliations:** 1 Transplant Research Center, Nemazee Hospital, Shiraz University of Medical Sciences, Shiraz, Iran;; 2 Department of Surgery, Faghihi Hospital, Shiraz University of Medical Sciences, Shiraz, Iran

**Keywords:** Jaundice, Elevated liver enzymes, Abdominal pain

## Abstract

Gastric duplication cysts comprise 2-7% of gastrointestinal duplications, rare congenital malformations that can be present at almost any part of the alimentary tract. They mostly present with gastrointestinal obstruction symptoms, ulceration, and painless hemorrhage. Symptoms include nausea, vomiting, and fullness sensation. Gastric duplications are mostly cystic in shape. Herein, we present a 58-year-old man with a gastric duplication cyst, 70×30×35 mm in size, with the initial presentation of abdominal pain, icterus, and elevated liver enzymes. The patient provided informed consent for this report.

## Introduction

Congenital alimentary tract malformations are rare developmental errors that can be present at almost any part of the gastrointestinal (GI) tract.^1^^,^^2^ They have been given several different names, including enterocystomas, enterogenous cysts, supernumerary accessory organs, ileum duplex, giant diverticula, and unusual Meckel diverticula. Gastric duplications, the least common among all duplications, constitute 2-7% of GI duplications and mostly present with GI obstruction symptoms, ulceration, and painless hemorrhage, mostly in early ages.^2^ Most cases of gastric duplication cysts suffer from nausea, vomiting, and fullness sensation as the semi-obstruction symptoms. Gastric duplications are mostly cystic as shown by a conclusive study done by Holcomb et al.^3^ who reviewed 96 patients with 101 duplications over 37 years and observed that 75 of the duplications were cystic and 26 were tubular. Duplications are mostly located in the greater curvature of the stomach and do not communicate with the gastric lumen.^2^^,^^4^^,^^5^ We describe a patient presenting with a gastric duplication cyst and the initial presentation of icterus. It is deserving of note that the cyst was positioned in the proximity of the gastric lesser curvature and as such exerted pressure on the portal vein and caused jaundice. Our literature review showed a paucity of data on the alimentary tract duplications initially presenting with icterus and elevated liver enzymes.

## Case Report

A 58-year-old man presented with long-standing postprandial abdominal pain (epigastric area) for 25 years. The pain had been misdiagnosed and managed as peptic ulcers with proton-pump inhibitors and H_2_ blockers with moderate improvement of the symptoms. Recently, he had developed on-and-off icterus, right upper quadrant abdominal pain, fever, nausea, and vomiting. He had previous abdominal ultrasound evaluations, which were unremarkable. No significant history was noted except exposure to chemical weapons during the Iran-Iraq war 24 years previously. On physical examination, the vital signs were normal and stable. The epigastric area was mildly distended, and a mass was only just palpable. Physical examination was otherwise normal. Laboratory work-up was remarkable for elevated liver enzymes and serum bilirubin, which were checked twice at a 24-hour interval:

● Serum glutamic oxaloacetic transaminase (SGOT): 135 and then 148

● Serum glutamic pyruvic transaminase (SGPT): 187 and then 173

● Alkaline phosphatase: 564 and then 520

● Total bilirubin: 7.8 and then 7.9

● Direct bilirubin: 3.4 and then 3.8

The patient’s plain abdominal flat and upright X-ray were normal. Abdominal sonography revealed a 5-cm ovoid cystic mass arising from the lesser curvature (near the antrum) of the stomach distending toward the portal vein. Color Doppler sonography of the common and proper hepatic artery and the portal vein was performed to evaluate the possibility of the luminal invasion of a cholangiocarcinoma or adenocarcinoma of the pancreas as differential diagnoses, which revealed reduced blood flow of the common hepatic artery and proper hepatic artery without any intraluminal lesion. 

**Figure 1:**
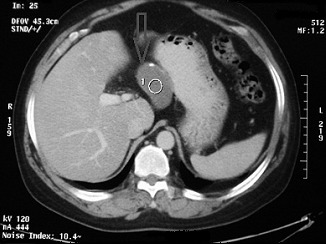
Abdominal computed tomography scan of the patient, revealing the duplication cyst in the proximity of the gastric lesser curvature.

Computed tomography (CT) scan of the lesion was compatible with the sonographic findings and showed a 70×30×35 mm mass with liquid density and thin calcification in the walls in the posterior aspect of the gastric antrum and pylorus in the vicinity of the posterior wall of the stomach (figure 1). The pancreas and other adjacent organs seemed to be normal.

**Figure 2:**
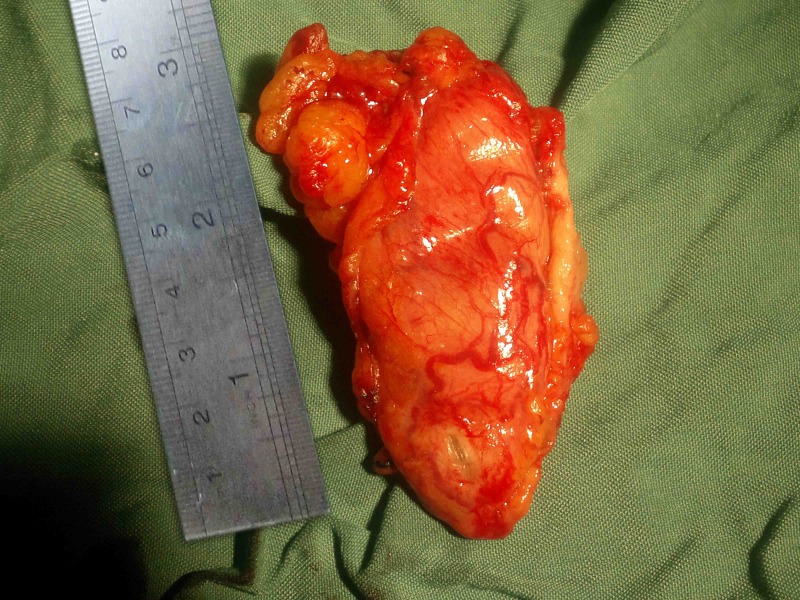
Gross appearance of the excised cyst.

**Figure 3:**
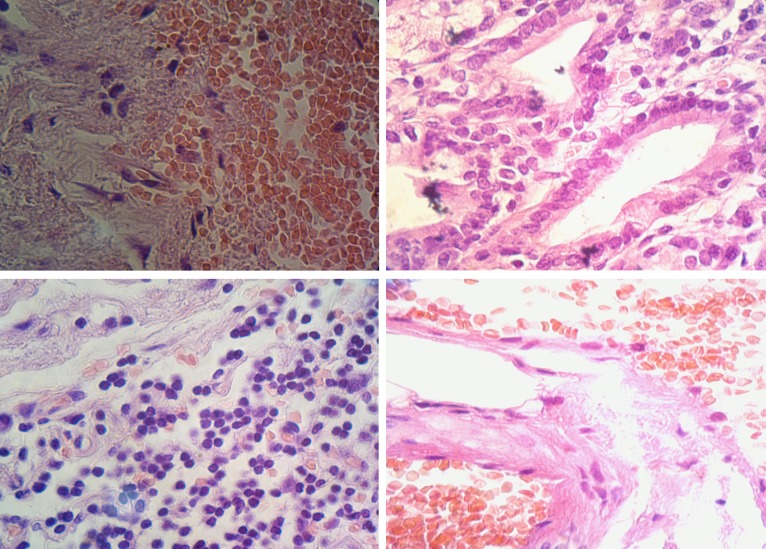
Microscopic appearance of the resected tissue.

The patient underwent exploratory laparotomy and excision of the duplication cyst. The cyst, as the abdominal CT scan reported, was located in the lesser curvature of the stomach, adherent to the stomach wall without any communication with the gastric lumen. The cyst stretched toward the portal vein, with obvious signs of inflammation in the area that caused a tension effect on the portal vein, resulting in the narrowing and flow impairment of the hepatic artery and common bile duct. The duplication cyst was excised successfully (figures 2 and 3).

The sample sent to the pathology lab was a small portion of the stomach, creamy-brown in color and measuring 7.5×3.5 cm in size, with a blind tip. Pathological diagnosis was gastric duplication as we had expected. The sample showed mucosal flattening with focal erosion, chronic inflammation with lymphoid follicle formation, and focal calcification of the wall. Surgical margins showed mild chronic inflammation with vascular congestion. Fortunately, there was no evidence of dysplasia or malignancy in the specimen. 

The patient had a normal uneventful postoperative recovery and was discharged 3 days after the operation with favorable results in one year of follow-up sessions without any of the symptoms with which he had initially referred.

The patient provided informed consent for this case report. 

## Discussion

Duplications are believed to be rare congenital malformations that can eventually occur at any part of the GI tract. In the Holcomb^3^ study with 101 cases of duplication, 21 duplications were confined to the thorax, 3 were thoracoabdominal, and 77 were abdominal.^3^

These malformations were first introduced and termed as GI duplications by Ladd^6^ in 1937. Since such anomalies are very rare, the current literature merely consists of few case reports. Among all abdominal duplications, gastric duplications are the least common (approximately 5%). They are more prevalent in men than in women, and patients present at a mean age of 3 years (One third are diagnosed during the neonatal period.) .Gastric duplications are cystic structures located along the greater curvature or posterior of the stomach. They typically do not communicate with the stomach cavity.^2^^,^^7^ Ultrasonography, CT, and myelography are helpful diagnostic tools.^3^

Our literature review demonstrated that the duplication of the alimentary tract has many different forms; therefore, the application of a single embryologic theory does not seem to be valid. This has led to the suggestion of some different theories to explain the embryologic events that result in duplications. 

Gastric duplications are rarely detected in adults since they present in the first years of life. In a study performed by Kremer et al.^8^ who presented 9 cases of gastric duplication, only one single case was adult.

Many duplications are incidentally diagnosed. However, most patients present with a combination of pain and obstructive symptoms. The symptoms may be the direct effects of the distention of the duplication or may be caused by the compression of the adjacent organs or blood supplies.^2^ Also, abrupt hemorrhage with hemodynamic instability can be seen when a cyst which is lined with the gastric mucosa ulcerates the adjacent organs or vessels.^9^

Obstructive presentations of gastric duplications are mostly epigastric postprandial pain and discomfort, nausea, vomiting, and abdominal mass.^5^ Some rare presentations include  hematemesis, gastrointestinal bleeding, recurrent pancreatitis, and perforation with peritonitis.^10^^,^^11^ In a case report by Kayastha et al.^1^ a gastric duplication presented as acute abdomen.

There is a paucity of information in the existing literature on the alimentary tract duplications initially presenting with icterus and elevated liver enzymes. What was also extremely rare as regards our patient was the fact that his cyst was located in the proximity of the gastric lesser curvature, creating a pressure effect on the portal vein and giving rise to jaundice.

Patients with undetected duplications may present with acute bowel obstruction or severe GI hemorrhage (in cases of ulcerating gastric mucosa within a duplication cyst). In these incidental situations, duplications should be surgically approached to avoid further complications. Surgical treatment of the alimentary tract duplications depends on the specific anatomical location of the lesion and its relation to the adjacent organs and vessels.^2^ In the Holcomb^3^ study, management was based on the patient’s age and condition, the location of the lesion, whether it communicated with the intestinal lumen, and the number of anatomic locations to which it was extended. It is essential that sufficient attention be paid to vital structures such as bile ducts and vessels during the excision of the cyst.

## Conclusion

The position of the duplication cyst in our patient was rare insofar as it was located in the proximity of the gastric lesser curvature. This uncommon position of the cyst had created a pressure effect on the hepatic vein and caused the incomplete obliteration of the common bile duct and the hepatic artery. As a result, the patient had developed icterus and elevated liver enzymes, which are deemed rare signs and symptoms of the initial presentation of the alimentary tract duplications. Furthermore, our patient had elevated alkaline phosphatase, SGOT, and SGPT as the late complications of a long-standing untreated gastric duplication. The post-hepatic signs and symptoms can be regarded as the complications of a gastric duplication cyst.
